# Pathways through organizational socialization: A longitudinal qualitative study based on the psychological contract

**DOI:** 10.1111/joop.12285

**Published:** 2019-07-17

**Authors:** Chris Woodrow, David E. Guest

**Affiliations:** ^1^ Henley Business School University of Reading UK; ^2^ King's Business School King's College London UK

**Keywords:** Socialization, Adjustment, Psychological Contracts, Pathways

## Abstract

In this study, we explore different pathways during organizational socialization through the lens of the psychological contract using in‐depth longitudinal qualitative methods. Analysis of 112 critical incident interviews with 27 newcomers across their first year of work reveals five distinct psychological contract pathways through socialization, within which fulfilment and breach influence adjustment by facilitating or restricting opportunities to learn and integrate, as well as influencing attitudes and behaviour. The analysis reveals that whilst perceived psychological contract fulfilment facilitates newcomer adjustment, perceived breach can disrupt the process. We provide a detailed account of the way socialization and the psychological contract unfold for newcomers over time, and show that psychological contract events can significantly alter the course of adjustment.

**Practitioner points:**

- Delivery of perceived promises that are of particular importance to newcomers during early tenure can accelerate adjustment. Managers should therefore attempt to find out which promised contributions are important to employees and prioritize their delivery.
- The negative effects of perceptions of broken promises on newcomer adjustment may be reversed by later delivery. Managers should explain the reasons for any broken promises and seek to fulfil them in the future.
- Ongoing support from managers can help newcomers to negotiate the difficult period after organizational entry, even where promises are perceived to have been broken.
- Direct managers should be made aware of information provided and promises made to newcomers by those responsible for recruitment.

## Background

Organizational socialization has been defined as ‘the process through which a new organizational employee adapts from an outsider to integrated and effective insider’ (Cooper‐Thomas & Anderson, 2006, p. 492), which is at its most intense in the initial weeks and months after entry (Van Maanen & Schein, 1979). Socialization has been characterized as a time of insecurity, during which newcomers attempt to cope with (Ellis *et al*., 2015) and reduce (e.g., Lester, 1987) stressful uncertainty.

The literature to date indicates that successful socialization results in adjustment, which involves developing sufficient knowledge, clarity, and confidence (Bauer, Bodner, Erdogan, Truxillo, & Tucker, 2007) about the new role, team, and organization to perform job requirements effectively (Haueter, Macan, & Winter, 2003); achieving acceptance from insiders (Moreland & Levine, 1982; Ostroff & Kozlowski, 1992); and developing stable and positive work‐related attitudes (Saks, Uggerslev, & Fassina, 2007). Current research has identified several factors that influence adjustment, including the methods used by organizations to facilitate socialization (Jones, 1986) and proactive newcomer behaviour (Saks, Gruman, & Cooper‐Thomas, 2011). The psychological contract, which develops during socialization (Rousseau, 1990), also drives employee attitudes at this time (e.g., Lapointe, Vandenberghe, & Boudrias, 2013).

We provide a detailed account of newcomers’ socialization experiences across their first year of work, based upon psychological contract theory and using intensive longitudinal qualitative methods. In doing so, we aim to contribute to the literature in two areas.

Existing studies have uncovered a variety of antecedents and consequences of adjustment, often based upon quantitative designs that imply a ‘stable set of forces that steadily push and pull on newcomers’ (Ashforth, Sluss, & Harrison, 2007, p. 6). Beyond this, several studies have identified some relatively short‐term issues that indicate non‐linear forms of adjustment, such as conflicts, using quantitative methods focusing on very early socialization (e.g., Nifadkar & Bauer, 2016) or qualitative methods with single data collection points (e.g., Gundry & Rousseau, 1994; Korte & Lin, 2013). What is lacking is a detailed understanding of the unfolding process of adjustment, and the dynamic processes that help to determine the effectiveness of socialization. If socialization is not always a smooth process, clearer understanding is required of the different pathways that newcomers experience across socialization, and the type of events that may act as potential turning points in these pathways. This research therefore explores pathways within socialization, utilizing an intensive longitudinal qualitative methodology. This approach allows detailed longitudinal exploration of individual journeys through socialization and specific emergent events that may affect the process. It also allows individual interpretations of and reactions to emergent events to be followed up and tracked over several time points.

To analyse pathways through socialization, we utilize the psychological contract. By focusing on psychological contract‐related events, we aim to contribute knowledge about how psychological contracts unfold and influence adjustment across socialization. Existing research indicates that perceived promises and breach mutually develop during socialization (e.g., De Vos, Buyens, & Schalk, 2003) and shape attitudes (e.g., Robinson & Rousseau, 1994). However, it is unclear how breach or fulfilment might affect adjustment, because studies have focused on attitudinal outcomes alone, rather than on learning or social integration. Moreover, little is known about the trajectories of individual psychological contracts across socialization. For example, it is possible that breach accumulates over time to an adjustment ‘tipping point’, where the addition of a particular breach on top of a series of previous breaches pushes individuals over a threshold and towards exit. Likewise, instances of fulfilment may accelerate adjustment or reverse a previous negative experience. In investigating these issues, we respond to calls for detailed research examining responses to psychological contract‐related events over time (Rousseau, Hansen, & Tomprou, 2018; Tomprou, Rousseau, & Hansen, 2015).

In summary, we contribute to the literature by identifying different pathways during socialization and exploring the dynamics of the psychological contract in this process, utilizing a distinctive longitudinal qualitative methodology. Below, an overview of the literature is provided, focusing on non‐linear forms of socialization, the psychological contract during socialization, and theoretical links between the psychological contract and adjustment, leading to the specific research questions to be addressed.

### Organizational socialization

There have been several conceptual approaches to the study of socialization. ‘Stage’ models split socialization into temporal phases (e.g., Jablin, 2001; Van Maanen, 1976), of which ‘encounter’, directly after entry, is considered most important. Other research has examined the antecedents of socialization and adjustment. The ‘tactics’ approach (Van Maanen & Schein, 1979) examines the organization's efforts to socialize newcomers, with formal and structured methods being most effective (Jones, 1986). Studies of learning show that those who acquire useful information during socialization (Bauer *et al*., 2007) and undertake proactive behaviours (Saks *et al*., 2011) report better adjustment.

Research has also examined non‐linear forms of socialization. First, studies have identified some relatively short‐term experiences that impact newcomer adjustment. Examples include conflict with co‐workers (Nifadkar & Bauer, 2016), acceptance of newcomers by insiders (Korte & Lin, 2013; Moreland & Levine, 2002), critical incidents that communicate cultural norms (Gundry & Rousseau, 1994), and early support or undermining (Kammeyer‐Mueller, Wanberg, Rubenstein, & Song, 2013). Unmet (Wanous, Poland, Premack, & Davis, 1992) and misinterpreted (Korte, Brunhaver, & Sheppard, 2015) expectations have been shown to hinder adjustment.

Second, newcomers can experience specific periods that are characterized by positive or negative attitudes during socialization. The honeymoon–hangover effect occurs where a job change engenders an uplift in satisfaction that subsequently decreases over time (Boswell, Boudreau, & Tichy, 2005). Satisfaction peaks are particularly high for those who learn more during socialization (Boswell, Shipp, Payne, & Culbertson, 2009), whilst institutionalized socialization tactics can reduce any decrease in satisfaction that occurs after this peak (Wang, Hom, & Allen, 2017).

Third, there has been some examination of discrete critical events that occur during socialization. Louis (1980) describes ‘surprises’ that occur when newcomers’ overly positive expectations meet with reality, precipitating sensemaking and information seeking. Lee and Mitchell's (1994) unfolding model of voluntary turnover begins with the occurrence of a ‘shock’ event, which may be perceived as negative or positive and internal or external to organizational life. This triggers a ‘script’ that can result in turnover during socialization (Holtom, Goldberg, Allen, & Clark, 2017; Kammeyer‐Mueller, Wanberg, Glomb, & Ahlburg, 2005).

In sum, existing literature has identified several factors that affect the socialization process, some indicating non‐linear forms of adjustment. However, research has not explored the nature and range of individual adjustment pathways that occur across socialization, and whilst it recognizes the role of events in shaping adjustment, it has yet to provide detailed insights into the way in which events can shape a dynamic socialization process. Our study seeks to investigate individual pathways across the entirety of the socialization process by addressing the following research question:

*Research Question 1*: What are the different pathways that are taken towards more or less successful socialization?

### The psychological contract during socialization

If different pathways are identified, this raises questions about their characteristics and what determines them. We examine these questions utilizing psychological contract theory. The psychological contract has been defined as ‘individual beliefs, shaped by the organization, regarding terms of an exchange agreement between the individual and their organization’ (Rousseau, 1995, p. 9). Several lines of research have examined the role of the psychological contract during socialization.

First, theoretical accounts have highlighted the importance of individual (e.g., information‐seeking behaviour) and organizational (e.g., overarching goals) factors in the development of perceived promises and breach during socialization (Morrison & Robinson, 1997; Rousseau, 2001; Rousseau *et al*., 2018; Shore & Tetrick, 1994), which lead to transactional or relational psychological contracts (Rousseau, 1990). Empirical research confirms that perceptions of promises (Robinson, Kraatz, & Rousseau, 1994) and the extent to which they are breached or fulfilled (De Vos & Freese, 2011) change across socialization, driven by individual differences, information acquisition, and existing organizational norms (De Vos, 2005; Thomas & Anderson, 1998).

Second, studies have demonstrated that psychological contract perceptions predict changes in perceived obligations and breach across the first year in the job. For example, employee perceptions of their own contributions and those of the organization positively predict levels of perceived employee and organizational obligations across the first 12 months of service (De Vos *et al*., 2003; Lee, Liu, Rousseau, Hui, & Chen, 2011). Additionally, newcomers perceive lower relational‐based employer obligations across their first 8 months, and consequently perceive greater breach and display poorer attitudes (Tekleab, Orvis, & Taylor, 2013).

Third, studies have examined the role of psychological contract perceptions in employee attitudes during socialization. Perceived breach can negatively affect trust, intention to remain, satisfaction, and turnover 2 years post‐entry (Robinson & Rousseau, 1994). Organizational commitment mediates the relationship between breach reported by newcomers and turnover intentions measured 4 months later (Lapointe *et al*., 2013). The attitudes of newcomers who experience breach may take several paths, including remaining below pre‐breach levels or improving beyond them, dependent upon the salience of breach, levels of organizational support (Solinger, Hofmans, Bal, & Jansen, 2016), and coping responses (Bankins, 2015). Perceived employee obligations around entry can also drive attitudes at 8 weeks (Delobbe, Cooper‐Thomas, & De Hoe, 2016). Additionally, psychological contract perceptions act as an intervening mechanism during socialization. Breach mediates the effect of leader–member exchange and perceived support on intention to leave measured at 6 months (Dulac, Coyle‐Shapiro, Henderson, & Wayne, 2008), whilst both perceived promises and fulfilment mediate the relationship between learning and attitudes across the first 3 months of tenure (Woodrow & Guest, 2017).

Existing literature indicates that the psychological contract, and particularly the formation and delivery of perceived promises, influences attitudes during socialization. However, research has yet to consider the effect of the psychological contract on other aspects of adjustment beyond attitudes. In addition, it remains unclear how the psychological contract unfolds across the entirety of socialization. This is because studies have generally used aggregate‐level quantitative methods, often with short follow‐up periods of 6 months or less (e.g., Delobbe *et al*., 2016; Dulac *et al*., 2008), rather than exploring the emergence of breach or fulfilment events and any affects across socialization, for which a 12‐month follow‐up period is considered necessary (Bauer, Morrison, & Callister, 2007). Our study therefore addresses these issues.

### The psychological contract and adjustment

Existing theory suggests that psychological contract‐related events may be key drivers of adjustment during socialization (e.g., Morrison & Robinson, 1997; Rousseau, 2001; Rousseau *et al*., 2018; Shore & Tetrick, 1994). At entry, newcomers hold underdeveloped psychological contracts derived from pre‐organizational experience, education, and promises arising from recruitment (Shore & Tetrick, 1994). After entry, newcomers absorb information from their environment during their first few weeks and months (Louis, 1980) in line with uncertainty reduction theory (Berger & Calabrese, 1975), which involves the communication of further promises. Some promises are made explicitly through written or verbal communication, whilst others may be more implicitly encoded via observing others or organizational communications (Rousseau & Greller, 1994). The psychological contract that is formed acts as a schema that guides newcomers’ behaviour (Rousseau, 2001). This remains relatively stable after the initial weeks and months of work, as a ‘maintenance’ phase is entered (Rousseau *et al*., 2018), although new promises may continue to be added (Rousseau, 2001).

Research suggests that ongoing organizational promise fulfilment facilitates some aspects of adjustment during the first year of tenure, with employees responding with their own contributions (De Vos *et al*., 2003) via the mechanism of social exchange, reflected in positive attitudes (Zhao, Wayne, Glibkowski, & Bravo, 2007). Importantly, it is also likely that some organizational contributions (e.g., training) allow employees to learn about their environment, and some employee contributions (e.g., undertaking specific tasks) involve immersion in organizational life. Consequently, fulfilment could lead to opportunities to build relationships, knowledge about the new environment, and positive work‐related attitudes, indicating adjustment.

Psychological contract breach, where the organization is perceived to have failed to deliver a promise, may derail this process. Breach acts as a ‘disruption’ to the psychological contract, prompting employees to reconsider the terms (Rousseau *et al*., 2018). Following social exchange, newcomers may remove some contribution after breach, accompanied by negative attitudes towards work (Zhao *et al*., 2007) and decreasing levels of trust in the organization (Robinson, 1996). In serious cases, employees may experience strong feelings of violation, an emotional reaction involving anger and frustration (Morrison & Robinson, 1997). The removal of some organizational contributions (e.g., support) may prevent opportunities to learn and socially integrate that are required for adjustment. Breach therefore has the potential to damage the developing employment relationship, leading to negative attitudes and behaviour, poorer social relationships, inhibited learning, and, potentially, turnover.

In sum, consistent fulfilment may lead to better adjustment across socialization, but breach events may disrupt this process. However, these issues are yet to be systematically examined in the literature. Existing studies have focused largely upon how the psychological contract affects newcomer attitudes rather than other aspects of adjustment. Additionally, studies have rarely used detailed exploratory methods that could shed further light on individual experiences during socialization across the first 12 months of tenure. Consequently, it remains unclear how long any effects of fulfilment or breach on adjustment last, whether they may be reversed, and what types and intensities of event are necessary to affect adjustment.

We therefore examine individual accounts of the psychological contract at regular 3‐month intervals across the first year of tenure in an inductive and exploratory fashion. In particular, we address the following research question:

*Research Question 2*: How do specific instances of perceived psychological contract breach and fulfilment affect adjustment within pathways through socialization?

Additionally, it is unclear how the timing of significant events during socialization affects adjustment. Robinson and Morrison's (2000) theory of psychological contract violation development suggests that perceived breach is particularly likely during very early socialization, when uncertain newcomers are vigilant to inconsistencies in the psychological contract and misunderstandings can easily occur between the two parties to the deal, especially if communication is inadequate (Morrison & Robinson, 1997). Newcomers are also unlikely to have built supportive relationships at this very early stage, which buffer against the negative effects of breach (Sutton & Griffin, 2004). This suggests that breach may be most common and damaging during very early socialization. On the other hand, very early tenure has been described as a time of promise negotiation (e.g., Shore & Tetrick, 1994), when the psychological contracts of inexperienced newcomers may more easily cope with deviations in the employment relationship compared to experienced staff whose psychological contracts have been formed over a longer period (Rousseau, 2001). This suggests that breach may be less harmful at an early stage of tenure. Since theory in this area is mixed, we explore the role of timing in the outcomes of breach and fulfilment by answering the following research question:

*Research Question 3*: Does the experience of breach and fulfilment, and any effect on adjustment within pathways, differ depending on when it occurs during the socialization process?

## Methods

### Design

The research, conducted in a large hospital in London (UK), adopted a longitudinal qualitative design. The study used repeated interviews, including critical incidents (Flanagan, 1954). This type of phenomena‐based case study provides a more suitable method to assess our exploratory research questions and build theory (Eisenhardt & Graebner, 2007) compared with more intensive methods such as diary studies. This approach also allowed the investigation of the experience of breach and fulfilment over time, enabling participants to reflect upon the same event at different points.

### Procedure

Newcomers to the organization were invited to participate in the study during 8 weekly ‘sign‐in’ days, where they reported to HR on their first day of work. Those who were happy to participate undertook an initial interview. Up to five interviews were conducted with each participant across the study, at entry, 3, 6, 9, and 12 months. Twelve months has been used as acceptable follow‐up period to capture the important aspects of socialization (De Vos & Freese, 2011). The remaining interviews were evenly spaced at 3‐month intervals to examine the evolving socialization process. This follow‐up schedule is considered appropriate for capturing the meaningful aspects of socialization (Bauer *et al*., 2007; De Vos *et al*., 2003).

### Participant characteristics

Forty‐one individuals undertook an initial interview at day 1. Of these, nine could not be contacted again, four were current employees of the organization, and one was joining on a short‐term contract, leaving a usable sample of 27 participants who undertook 112 interviews. Eight participants voluntarily left the organization during the study period. Participants were a mix of occupational groups broadly in line with that of the workforce as a whole, including nurses, other health professionals, and administration staff. Six participants were entering their first job in their profession. The mean age of participants was 31 years, 71% were female, and 38% were from Black, Asian, and Minority Ethnic backgrounds.

### Interviews

Each interview lasted up to 40 min. The day 1 interview was used to collect demographic data and examine perceived psychological contract‐related promises and commitments that the organization had made. Each subsequent interview began with a series of questions examining perceived learning, attitudes, social integration, supervisory, and other support. Psychological contract breach and fulfilment were assessed in two ways. A critical incident technique (Flanagan, 1954) was used, with participants asked to recount any incidents that related to negative or positive events at work. These were probed if they reflected perceived breach or fulfilment. Additionally, participants were asked about the extent to which managers were perceived to have kept or broken any promises or obligations, and whether they felt they had been treated fairly, with emergent issues probed. Participants were also asked about the status of any perceived breach and fulfilment described in previous rounds.

### Analysis

NVivo software was used to organize transcripts and record coding. Analysis began with thematic coding of individual interviews using the approach of Miles and Huberman (1994). At stage 1, data were coded specifically for instances of perceived breach, fulfilment, organizational promises, adjustment, and attitudes. For the purposes of analysis, ‘perceived promises’ were coded where employees believed that their organization had promised or was obliged to provide a particular contribution in the future, in line with common conceptualizations (e.g., Coyle‐Shapiro & Kessler, 2002; Rousseau, 1990). Perceived breach was defined as ‘the employee's perception regarding the extent to which the organization has failed to fulfil its promises or obligations’ (Zhao *et al*., 2007, p. 649). Perceived fulfilment was coded where participants demonstrated the cognition that their organization had fulfilled promises or obligations. Codes relating to adjustment were applied where participants discussed learning about the role, team, and organization that enabled effective job performance (Haueter *et al*., 2003), the development of positive work‐related attitudes (Saks & Ashforth, 1997), and positive relationships with insiders (e.g., Moreland & Levine, 1982; Ostroff & Kozlowski, 1992). A broad view of learning was used which included aspects of social integration and acceptance, in line with previous conceptualizations (e.g., Ostroff & Kozlowski, 1992).

At stage 2, exploratory open coding was applied to the entire data set to build upon preliminary codes and develop new ones. This initial coding process led to the development and refinement of lower level codes, which were grouped into pattern codes (e.g., ‘perceived causes of breach’, ‘perceived consequences of fulfilment’, ‘job learning’). At this stage, a set of perceived types of breach and fulfilment experienced by study participants was extracted from the data (see Table 1). A sample of approximately 20% of transcripts were independently examined for critical events by two coders. There was minor disagreement on a small number of events, which was resolved by discussion and used to inform ongoing coding.

**Table 1:** Breach and fulfilment experienced by study participants

Type of breached/fulfilled promise	Content of breached/fulfilled promise
Training	To provide necessary training
Inadequate staffing	To provide adequate staffing to enable optimal newcomer performance (staffing)
Support structure	To support newcomers via mentoring, induction, or appraisal (support)
Pay and advancement	To promote a newcomer, pay them a particular salary, or provide a salary increment (pay)
Equipment	To provide equipment necessary to perform the job
Role rotation	To provide a particular training role rotation at a particular time (rotation)
Workload	To allocate a particular level of workload or number of hours
Quality of care	To support staff in providing an adequate standard of quality, or to maintain a particular level of service quality (quality)
Time off	To provide reasonable time off at an agreed time

Terms in brackets denote abbreviations used in Tables 2 and 4.

At stage 3, data were analysed on a longitudinal basis using the systematic approach of Saldaña (2003), which involves examining data using three sets of analytical questions. ‘Framing’ questions are used throughout the coding process and identify changes over time (e.g., Which differences occur between data collection points; when do these occur?); ‘Descriptive’ questions act as a bridge between framing and interpretation (e.g., what increases or is cumulative across time; do ‘surges’ or ‘epiphanies’ occur?); ‘Analytic/interpretive’ questions are then used to interpret the changes identified earlier (e.g., are there interrelationships between changes?).

Interviews were first analysed on a within‐participant, case‐by‐case basis, in conjunction with codes developed earlier. At this stage, various changes over time were coded (e.g., ‘breach of salient promise followed by intention to exit’; ‘over‐fulfilment followed by learning about role’), each with associated codes from stages 1 and 2 (e.g., ‘social integration’; ‘learning’; ‘support’). Between‐participant analysis was then undertaken. This resulted in the emergence of five overarching themes concerning distinct pathways through socialization (e.g., ‘Gradual adjustment through promise fulfilment’; ‘Accelerated adjustment through over‐fulfilment’), each underpinned by a set of codes relating to aspects of the employment relationship (e.g., ‘breach salience’; ‘management support’). Analysis was aided by the use of Miles and Huberman's (1994) time‐ordered matrices, detailing each participant's journey using one entry for each time point. Table 2 shows the matrix for perceived breach events.

**Table 2:** Time‐ordered matrix for emergent breaches by follow‐up

ID	3 months	6 months	9 months	12 months
3	Staffing		Time off Pay	
4	Training	Staffing Training Time off	Time off Pay	
5	Support	Training		
6	Pay		Training	
7	Staffing Support		Pay Training	
8				Workload
10	Staffing	Training		
12	Staffing			
14		Rotation Workload	Training	
16	Equipment	Quality		
18				
19				
21	Support Training	Quality	Training	
22	Support Training Rotation			
23	Support			
24	Support		Staffing	
26	Workload Support	Workload Support		
28	Support			
30			Equipment	
31	Support			Workload Support Pay
33	Training Time off	Support	Pay	
34			Staffing	
36	Support			
37				
38	Support	Quality		
39	Support			
41	Training	Staffing		

### Findings

#### Types of breach and fulfilment

Initial analysis of the entire data set identified nine distinct types of promise that were breached or fulfilled during the study (shown in Table 1). Some related to promises that had emerged at the initial interview, whilst others had subsequently become part of the psychological contract. Promises were derived from a variety of sources, but the agent of delivery (Rousseau, 1995) was in almost every case viewed as being a direct supervisor or a departmental supervisor with specific responsibility for training, who was often not the source of the initial promise. Some participants (such as those described in pathway 4) altered their attribution of responsibility for breach from hospital management in general to their direct manager as the study progressed.

Instances of fulfilment relating to specific promises emerged less often than instances of breach, because participants often described fulfilment in more general terms. For example, Participant 19 stated that ‘I can't think of anything that … they've promised and it's not been delivered’. Most commonly reported instances of breach across all rounds and participants related to *Training* (12) and *Support Structure* (15). A greater proportion of study participants (20 of 27) reported breach at 3 months compared to 12 months (2 of 19). Most participants did not report new instances of breach or fulfilment at every follow‐up, and three individuals did not report any breach during the study.

#### Initial psychological contracts

The initial interviews focused upon perceived promises made at entry. The following types of promise were reported at this stage: *Support Structure*;* Training*;* Workload; and Quality of Care*. In line with Rousseau (1995), promises originated via both human (e.g., conversations with insiders during recruitment, including but not limited to managers) and structural (e.g., organizational literature) sources. Some were more implicit, such as the provision of particular types of training that were described in literature but never formally promised. Others, such as the provision of a mentor, were promised explicitly during recruitment. Initial interviews were conducted prior to individuals having started their jobs, and many stated that they expected to learn more about what the organization would provide during their formal induction and initial weeks in post.

#### Adjustment

Many participants discussed adjustment in terms of the development of a strong network in their work area and generally positive attitudes towards their work. Less experienced newcomers, particularly those entering their first job in their profession, described developing an understanding of their role as an important part of adjustment. For more experienced newcomers entering from similar jobs elsewhere, the role itself was far less important. An experienced midwife illustrates this:

I know the midwife area is always busy….midwifery is midwifery, no matter where you find yourself. [Participant 33]

Several saw their ability to integrate through proactive behaviour as a major achievement. Newcomers highlighted the importance of social integration into their work area, rather than into the organization as a whole:

I guess, if anything, I feel more part of this building and the ethos of the building…I don't feel like I am part of the hospital staff. [Participant 16]

#### Pathways of breach and fulfilment through socialization

Longitudinal analysis of the data set led to the emergence of five distinct psychological contract pathways through socialization, each exhibiting distinct temporal characteristics. Additionally, three themes emerged that cut across the pathways, relating to manager support, organizational responsiveness, and the type of promise made. These pathways are represented graphically in Table 3 and explained below. Table 4 summarizes the critical psychological contract events reported for each pathway.

**Table 3:** Six psychological contract pathways through socialization

	Pathway	Local support	Organizational responsiveness	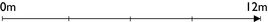				
1	Gradual adjustment through promise fulfilment	Strong	Strong	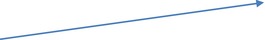				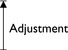
2	Accelerated adjustment through over‐fulfilment	Strong	Strong	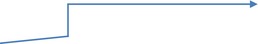				
3	Positive turning point through fulfilment	Mixed	Mixed	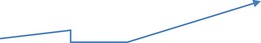				
4	Cumulative breach to tipping point	Weak	Weak	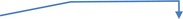				
5	Negative turning point through violation	Weak	Weak	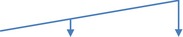				

**Table 4:** Summary of psychological contract events reported for each socialization pathway

Pathway (*n*)	All breach events (*n*)	All fulfilment events (*n*)	Turning point incidents (follow‐up)
1. Gradual adjustment through promise fulfilment (13)	Equipment (2) Staffing (4) Pay (1) Quality (1) Rotation (1) Support (4) Training (4) Workload (3)	Time off (1) Pay (1) Equipment (1) Rotation (2) Staffing (1) Support (3) Training (6)	N/A
2. Accelerated adjustment through over‐fulfilment (2)	Pay (1) Training (1)	Equipment (1) Pay (1) Support (1) Training (2)	Training (T1) Training (T1)
3. Positive turning point through fulfilment (2)	Staffing (1) Pay (1) Support (2) Time off (1) Training (1)	Support (1) Training (2)	Support (T2) Training (T3)
4. Cumulative breach to tipping point (2)	Staffing (1) Pay (1) Quality (1) Support (2) Training (1)	Quality (1) Training (1)	Quality (T2) Pay (T3)
5. Negative turning point through violation (4)	Staffing (2) Pay (2) Rotation (1) Support (3) Time off (3) Training (3) Workload (2)	Support (2) Training (1)	Support (T1) Support (T2) Time off (T3) Time off (T3)

T1 = 3 months; T2 = 6 months; T3 = 9 months.

##### Pathway 1: Gradual adjustment through promise fulfilment

The first emergent pathway involved individuals for whom adjustment happened gradually and reasonably smoothly, with participants reporting that promises had generally been fulfilled throughout. This was the most common pathway and included 13 study participants (around half the sample).

Most participants, and particularly those more experienced in their profession, viewed promise delivery with a sense of ambivalence. When asked about the delivery of promises and obligations, statements such as ‘I can't complain’ [Participant 34] were common. Hence, in many cases, fulfilment acted via the removal of potential barriers to adjustment. Some participants described the fulfilment of promises, such as provision of training that had been discussed at interview or described in a job advertisement. Others stated that little explicit promise‐making had taken place. For some less experienced participants, fulfilled promises contributed to adjustment by making contributions (e.g., training or enlarged job content) that enabled them to learn the important aspects of their new environment and develop social relationships. Participant 5, a nurse, was made promises regarding training and support. Delivery of these promises helped her to integrate socially, and to learn about her role and pass a test necessary to administer drugs:

I passed first time…other colleagues who'd already done the test were very supportive. My practice development nurse is very supportive….it is something you will have had to have passed before you can administer drugs.

Most participants in this pathway reported broadly positive attitudes throughout, demonstrating adjustment after 6 or 9 months. Accounts were characterized by strong support from their direct managers who responded to minor instances of breach. Many reported that some promises had not initially been fulfilled. However, these often related to issues that were not of particular importance to the individual and were swiftly righted by the organization. For example, Participant 23, an allied health professional, stated at 3 months that a promised supervisory structure was not in place:

When I first started, they weren't really sure who even my supervisor was going to be, and I think those processes took some time. I didn't have an appraisal until two months into my first job.

However, at later follow‐ups, she reported that this situation had little effect on her attitudes, since it was not of particular importance to her:

I've got a new supervisor now… absolutely fine, no problem at all now.

[I am] really integrated … I suppose it takes a few months to settle in and feel like ‘okay, now I am part of the team’.

##### Pathway 2: Accelerated adjustment through over‐fulfilment

The second pathway that emerged concerned cases where fulfilment acted as an event that led to a sharp upturn in adjustment. The defining characteristic of these cases was the presence of over‐fulfilment, where perceived promises were delivered above the expected level.

This type of turning point was experienced by two individuals. In each case, it was reported at 3 months and concerned the provision of training. This experience was viewed as an act of goodwill towards newcomers that increased trust in the organization and enabled them to quickly build social networks and learn about their environment, setting the course for a positive journey through socialization. Subsequently, each participant adjusted quickly to their role, reported a general happiness with most aspects their work at each additional follow‐up, and remained with the organization at 12 months.

Participant 18, a non‐clinical support employee, reported at the 3‐month follow‐up that she had been provided with training over and above what had been promised:

A lot more… it's just the training I've received …I've never done dental before, but I've received quite a lot of support….I'm extremely happy.

At the 6‐ and 9‐month interviews, she felt fully adjusted and reported that promises were still being met:

I was very positive and I still am. Things are getting even better and better…I get quite a lot of support, which is excellent….I've been very lucky that in my case they've kept [their promises], especially with the training and the support…I got it all.

I have integrated really well… in terms of my job and the other departments.

Participant 6, a nurse, stated at the initial interview that she had been promised ‘training and….in the first months, I will be very well supported’. At 3 months, she revealed that this training had been above the promised level:

Better than I thought it would be. I had more support than I thought I would have…they had somebody to work with me for my first few shifts …it is nice to have somebody just to show you how they do things.

At subsequent interviews, she continued to report positive attitudes, strong line manager support, good knowledge of her environment, fulfilled promises, and positive relationships. At 12 months, she reported feeling adjusted and socially integrated, particularly within her immediate work area:

I think [I am integrated] more in my job on the unit than at [the hospital] as a whole. …I feel I'm integrated enough at [the hospital] so it's fine.

##### Pathway 3: Positive turning point through fulfilment

The third emergent pathway was characterized by a negative experience that, through organizational action, later became positive and led to adjustment. The two individuals in this pathway experienced the breach of a salient promise. However, at subsequent interviews they revealed that the organization had fulfilled this promise, which led to a reversal of their negative attitudes and perceptions of adjustment.

The initial breach in these cases was reported at 3 months, with subsequent organizational action to remedy it reported at 6 or 9 months. One breach related to a promised support structure and one to promised training. Line manager support and organizational responsiveness were intertwined and of critical importance to this pathway, since a change of supervisor led to delivery of a perceived promise that the previous supervisor had failed to deliver in both cases. The effect of promise delivery provided an uplift in attitudes and allowed participants to learn more about their environment and build networks via the actual contribution offered, resulting in better adjustment.

Participant 33, an experienced midwife, stated at entry that she joined the organization in order to undertake a particular training course that was described in messages from the organization. At 3 months, she reported beginning to settle into the role, aided by previous experience. However, she reported a breach when a supervisor stated that it was not possible to undertake the training after all, leading to relationship difficulties with the supervisor and inhibiting learning. At this stage, the participant considered leaving:

I spoke to [the supervisor] about a course that I wanted to do… she said there's no place for me…it's one of my main reasons for coming….I am thinking about whether to stay here and continue to wait for me to do the course or to try somewhere else.

At 9 months, the situation changed. Her supervisor had been replaced by an individual who found her a training place:

[The supervisor] has been away for some time now, and somebody else is doing her job… [they will] help me go on the course….I don't think I have any problems as far as work is concerned.

The delivery of this promise, coupled with the change in supervisor, triggered far more positive attitudes about the environment and team:

I've been there for about nine months now at least, I've become used to the routines of the hospital, familiar with the hospital policies, so I feel much at home …they've all been nice, the managers and my colleagues have been nice.

Finally, at 12 months, she reported feeling fully adjusted and integrated, having seen a change in the way the department operated since joining:

When I first started the newcomers were isolated, but I've really seen a change…the initial welcome was not the best….but things have really improved.

##### Pathway 4: Cumulative breach to tipping point

The fourth pathway involved a negative turning point in the form of a psychological contract breach. We classify this as a ‘tipping point’, whereby one particular breach following a series of breaches pushed individuals over a threshold, leading to exit.

Two individuals in the data set were coded under this pathway. In each case, a number of breaches were experienced until, finally, the newcomers decided that they no longer wished to remain in the organization. One individual left for another job between 6 and 9 months of service. The second was searching for a new post at the 12‐month interview. Adjustment was stunted throughout, because breaches denied access to aspects of organizational life that would promote learning and social adjustment, causing frustration and impaired trust in management. Both instances of tipping points occurred during the later phases of the study, at 6 and 9 months, relating to ‘quality of care’ and ‘pay and advancement’. These pathways were characterized by perceptions of poor line manager support, a large number of breaches during socialization, and a lack of organizational resolution of these breaches.

Participant 7, a trainee allied health professional, experienced a difficult time during his first year of work. At 3 months, he learnt that there was a recruitment freeze and his department was likely to close, and reported breaches relating to understaffing and support. Despite some setbacks, he reported beginning to adjust through learning about his new organization and displayed generally positive attitudes:

It's going alright….they've given me training and made sure that I'm alright…the biggest achievement really is being able to integrate myself into all the departments.

At 6 months, these breaches had not been resolved, and a further breach emerged relating to removal of training opportunities. The final straw for this participant, however, was revealed at the 9‐month interview, concerning a promised salary increment that had been reneged upon:

You are supposed to get a pay increase after six months, and we were promised to get this, and then all of a sudden they turned around and said: ‘you can't have it unless you do this [training] program’.

This was one broken promise too many, and he began searching for another job, continuing at the 12‐month interview. At this point, he stated that whilst he felt somewhat socially integrated at the team level, he had a poor relationship with his supervisor and the breaches relating to training had prevented adjustment into the role:

Everyone is friendly with literally everyone at work…I am friends with everyone except my line manager…[but] I haven't officially learned anything, because I haven't been able to go on any training courses. CV wise, I haven't actually had any advances.

This participant attributed the breaches at 3 months to organizational‐wide issues rather than to his own manager, describing them as ‘a bit unfair, but…unfortunately a necessary evil’. The later breaches, by contrast, were attributed to his direct manager: ‘I'm not sure it's so much saving money; I think that was more of a way to try and get to us a little bit’.

##### Pathway 5: Negative turning point through violation

The final pathway that emerged from the data also involved a negative turning point in the form of a psychological contract breach. Here, four participants who were experiencing an otherwise broadly positive socialization experience were jarred towards poor levels of adjustment by the perceived breach of a particularly salient promise. In each case, the breach led to job search behaviour. In two of these cases, the individuals left the organization for another job before 12 months of service. Breaches of this type elicited strong feelings of anger, frustration, or betrayal, characteristic of high levels of psychological contract violation (Morrison & Robinson, 1997). This type of critical turning point event was reported at varying follow‐up points: at 3 months, at 6 months, and twice at 9 months. The types of promise to which breaches pertained also varied. Two related to supervision and two to access to annual leave. The critical issue in these cases was the importance of the promise to the individual.

Participant 4, a trainee health professional, was assured at entry that she would be able to take a holiday at a particular time. During the early interviews, minor breaches were reported, relating to inadequate staffing, time off, and access to training. She continued to adjust, although she reported a poor relationship with her manager, stating at 6 months that she had learnt about her new environment and role: ‘I feel completely a part of the hospital because the people who work here are amazing…except my manager’; and that ‘nothing is perfect, but for now…I'd say I'm happy’. However, at 9 months, she reported that the promise to take holiday had been reneged upon by her manager. This elicited a strong negative reaction and, consequently, she reported that her positive attitudes towards the hospital were reversed and began searching for a new job:

When they hired me…they said ‘no problem’. Now because they are doing cuts they've come to us and said ‘now you can't’. For me that is definitely not acceptable… So I already told my boss that if I find something else …I will leave.

If they start making rules when only one can go on holiday it's wrong, which was not part of the deal when they hired me! Those things don't make me feel so connected to [the hospital].

Participant 26, a patient‐facing administrator, experienced a similar journey. At 3 months, she stated that she was integrating well into her team and being given time by her mentor to learn the ropes:

[My mentor has] done it in stages with me, which is really, really helpful…she's given me the time to understand what I need to understand. And as I grasp it we move onto another part.

However, at 6 months, she reported two breaches which led her to begin searching for a new job. One related to management's failure to protect her from patient abuse and the other to the removal of a promise that staff could leave their shift once cover is in place:

The nurses, once they've done their handover, even if it's five past eight, they can actually leave once their cover has come in. And our line manager said the same thing. Well, once confronted by their manager, they've backed down. For me, I just think it's just so wrong.

These incidents were viewed as a serious betrayal, leading to anger and a poor relationship with her managers, and prompting her to state that ‘I'm not going to stay. That's not the sort of environment I can work in’. At the 9‐month follow‐up, she had left for another job.

The pathway was characterized by a departmental management structure that was perceived to be poor and unsupportive. Participants described a lack of responsiveness to complaints about breach, with attempts to discuss issues with managers often ignored. Individuals described feeling somewhat adjusted, having worked hard to build networks and learn about their environment, despite negative relationships with management. However, the breaking of a particularly salient promise derailed these efforts, setting them on a path to exit.

## Discussion

We present research designed to advance the literature on organizational socialization by addressing three interrelated issues that have remained unresolved. Because of the exploratory nature of our research questions, we undertook a longitudinal qualitative study using critical incidents of psychological contract breach and fulfilment to examine how newcomers interpret their experiences, and any specific events that occur, across the entirety of socialization.

Our first research question asked whether it is possible to identify pathways through the socialization process. Analysis led to the identification of five distinct pathways. The second research question addressed the role of psychological contract breach and fulfilment as a means of understanding and explaining adjustment within pathways. Analysis reveals that each of the five pathways was defined by psychological contract‐related characteristics. One pathway shows that many experience gradual adjustment, with promises generally fulfilled. Another reveals that exceeded promises can provide a positive turning point, accelerating adjustment. A third provides evidence that after breaches have occurred, subsequent fulfilment of salient promises can act as a positive turning point. A fourth reveals that newcomers can reach a ‘tipping point’ after a series of breaches, and a fifth shows that breach acts as a negative turning point where it involves violation of a salient promise. Our third research question concerned the timing of relevant events during socialization. Analysis confirms that events had a profound effect on the success of socialization at any time over the 12 months of our study, although some trends linked to particular pathways emerged. Over‐fulfilment that led to very rapid adjustment occurred in early socialization. Fulfilment of a previously breached promise that led to an upturn in adjustment occurred later in the process. ‘Tipping points’ that occurred after a series of previous breaches also occurred later in the process.

### Theoretical implications

Our findings demonstrate that psychological contract breach and fulfilment act as discrete ‘turning point’ events across the entirety of socialization, influencing adjustment and helping to determine the pathway that is taken through socialization. Whilst research has shown that socialization does not proceed linearly (e.g., Boswell *et al*., 2005), we add to the literature describing potential ‘issues’ (e.g., Korte *et al*., 2015) or ‘shocks’ (e.g., Holtom *et al*., 2017) that may occur. Socialization has been characterized as a process of overcoming challenging hurdles (e.g., Ashforth *et al*., 2007) that leads to sensemaking (Louis, 1980) and adjustment. This research shows that perceived breach and fulfilment of the psychological contract precipitate rapid adjustment as well as disengagement, such that many who go on to become socialized have experienced events with negative and positive outcomes. The psychological contract has often been positioned as an outcome of socialization (e.g., De Vos & Freese, 2011) or a driver of attitudes (Robinson & Rousseau, 1994). We show that it is important for all aspects of adjustment. Importantly, adjustment often hinged upon one particular aspect of the job, such as a training place. Hence, individuals who reported making efforts to integrate and build networks were still jarred towards poor adjustment (and possibly exit) when denied something of particular importance.

The findings of this study demonstrate that the function of breach and fulfilment may differ by stage. During early socialization, in what is often called the encounter phase, over‐fulfilment spurred employees into early adjustment by providing the tools to socially integrate and learn the job, facilitating positive attitudes. Fulfilment later in socialization sometimes served as corrective action to restore trust and aid adjustment that had been harmed by previous negative experiences. Breach that occurred late in socialization, particularly if following earlier breach, tipped some participants towards exit. Serious breach that resulted in strong feelings of violation acted primarily by impairing attitudes and trust in the organization, acting as turning points at any stage. Additionally, participants’ levels of previous experience influenced the importance of features of adjustment. Newcomers with limited experience in their profession were particularly concerned with role‐related aspects of adjustment, whereas experienced newcomers were more concerned with social integration, adding to previous findings that more uncertain inexperienced newcomers benefit more from the use of ‘institutionalized’ socialization tactics (Saks *et al*., 2007).

Our findings also provide several theoretical implications regarding the ‘unfolding’ nature of particular promises and their fulfilment or breach, an area that has received limited prior attention (e.g., Conway & Briner, 2005; Rousseau *et al*., 2018). First, previous theoretical work suggests that the effect of perceived breach can depend upon the importance of the breached promise (Morrison & Robinson, 1997; Schalk & Roe, 2007). We provide empirical evidence that perceived breach of particularly important promises affects adjustment. Second, previous cross‐sectional research has demonstrated that the effect of perceived breach on attitudes is not linear but becomes stronger when a certain intensity of breach is perceived (Rigotti, 2009). We add to these findings by showing that individual instances of breach may be additive over time in terms of their effects on all aspects of newcomer adjustment. Third, Tomprou *et al*. (2015) discuss the importance of perceived organizational responsiveness in repairing broken promises, and empirical evidence demonstrates that post‐breach support can enhance recovery with respect to commitment (Solinger *et al*., 2016). This analysis demonstrates that fulfilment of a previously breached promise may provide a positive turning point. Finally, previous research shows that over‐fulfilment may have a positive impact, albeit to a lesser degree than the negative impact of under‐fulfilment (Conway & Briner, 2002), and may lead to a positive renegotiation of the deal (Rousseau *et al*., 2018). Our research shows that in very early tenure, where organizational contributions refer to an issue that can aid learning about the environment, over‐fulfilment may have a very strong positive impact upon adjustment.

### Practical implications

This research has implications for the management of newcomers during socialization. First, the research implies that the management of broken promises is particularly important, because a series of unresolved breaches, even if seemingly unimportant when viewed individually, can inhibit adjustment and precipitate exit. Second, the research shows that damage done by broken promises may be undone through subsequent fulfilment. This suggests that where individual promises are broken, managers should not forget about them, since employees are unlikely to. Rather, managers should seek to explain why the promise was broken and try to fulfil it later. Our findings highlight the important role of the supervisor and the support they provide, since instances emerged where fulfilment of a previously breached promise was delivered by a newly appointed and more supportive manager. This points to a case for training supervisors in management of newcomers, sensitizing them to many of the points raised including the importance of managing the psychological contract.

Third, our research shows that delivery (or over‐delivery) of perceived promises concerning important learning opportunities during very early socialization can have strong and lasting effects upon adjustment, whilst other promises were viewed as less significant even when broken. This suggests that managers might prioritize particular types of contribution during early tenure, whilst being aware of relevant previous experience of newcomers. Finally, perceived promises may arise from a variety of sources during recruitment, of which managers in departments may be unaware. It is therefore important only to make promises that can realistically be kept, and for managers to be made aware of the perceived promises that newcomers may hold.

### Limitations and future research

Our study was conducted with a fairly small number of staff in one context. Whilst our aim was to elicit rich information about socialization, it is not clear whether the five pathways identified here are found in other contexts. Furthermore, three pathways each contained only two participants. Further research can confirm these or other potential pathways. Additionally, we only assessed the perceptions of newcomers, when the perceptions of organizational insiders who may play a role in both adjustment (Moreland & Levine, 1982) and the psychological contract (Guest, 1998) could provide an additional perspective.

Aspects of the emergent findings could be investigated further with longitudinal qualitative or possibly quantitative methods. One concerns the accumulation of psychological contract breach, where a particularly important question relates to how much breach individuals can take, over time, before they reach a tipping point. Second, the findings concerning subsequent fulfilment of a previous breach are novel. Future research might explore factors that facilitate this, such as the influence of a change of manager which was important here. Third, this research shows that the type of promise is important, and this issue may be usefully investigated in other contexts with more participants. Fourth, our study took place in a large hospital which may help to explain a gap between promises made during recruitment and some of the challenges of ‘local’ socialization, as well as the limited interest in integration into the organization as a whole. Future research in smaller organizations may present a different picture. Finally, our research highlights the general utility of adopting relatively novel methods to explore and build on theory, and specifically the use of longitudinal qualitative research to analyse the time domain in the evolution of the psychological contract and socialization.

### Conclusion

Our research advances knowledge about organizational socialization and the psychological contract in several ways. First, it has drawn attention to the variety of pathways in socialization. Second, it has demonstrated the value of the psychological contract in understanding the evolution of the socialization process, revealing that whilst it can be a smooth process, instances of fulfilment and breach can lead to turning points and tipping points. Longitudinal qualitative methodology advances our understanding of the dynamic state of the psychological contract during periods of instability such as socialization by using the critical incident technique to demonstrate how specific events affecting fulfilment or breach can alter the trajectory of organizational socialization. Rather than representing an outcome of socialization, the psychological contract can have a large influence on whether adjustment occurs at all.
